# Efficacy and Safety of Generic Sofosbuvir Plus Daclatasvir and Sofosbuvir/Velpatasvir in HCV Genotype 3-Infected Patients: Real-World Outcomes From Pakistan

**DOI:** 10.3389/fphar.2020.550205

**Published:** 2020-09-02

**Authors:** Saima Mushtaq, Tayyab Saeed Akhter, Amjad Khan, Aamir Sohail, Arshad Khan, Sobia Manzoor

**Affiliations:** ^1^Department of Healthcare Biotechnology, Atta-ur-Rahman School of Applied Biosciences, National University of Sciences and Technology, Islamabad, Pakistan; ^2^Centre for Liver and Digestive Diseases, Holy Family Hospital, Rawalpindi Medical College and Allied Hospitals, Rawalpindi, Pakistan; ^3^Department of Pharmacy, Quaid-i-Azam University, Islamabad, Pakistan; ^4^Department of Medicine, Karachi Institute of Medical Sciences, Combined Military Hospital, Karachi, Pakistan; ^5^Department of Neurosurgery, Medical Teaching Institution, Lady Reading Hospital, Peshawar, Pakistan

**Keywords:** hepatitis C, daclatasvir, sofosbuvir, velpatasvir, sustained virological response

## Abstract

**Background:**

Direct-acting antivirals (DAAs) therapeutic regimens are highly effective against chronic hepatitis C virus (HCV) infection. However, HCV patients with genotype 3 (GT3) respond in a suboptimal way. This study aims to identify which of the DAAs-based therapeutic regimens are the best option for GT3.

**Methods:**

Multiple governments and private tertiary care hospitals were involved in this real-life study of HCV-GT3 patients treated with DAAs. The efficacy and safety of generic sofosbuvir+daclatasvir±ribavirin (SOF+DCV±RBV) and sofosbuvir/velpatasvir±ribavirin (SOF/VEL±RBV) were assessed under the National Hepatitis C Program of Pakistan.

**Results:**

Out of 1,388 participants, 70% of patients received SOF+DCV in government tertiary care hospitals and 30% received SOF/VEL in private tertiary care hospitals. The overall sustained virological responses (SVR) was 95.5%. The SVR rates at 12 weeks were comparable between SOF+DCV (94.4%) and SOF/VEL (94.7%) in chronic HCV patients. However, The SVR rates at 24 weeks were high in cirrhotic patients treated with SOF/VEL+RBV (88%) then SOF+DCV+RBV (83%). Non-responders were high in SOF-DCV than SOF-VEL (4.1 *vs* 3.8%, *P* = 0.05) regimen. In multivariate models, the significant predictors of non-SVR were age >60 years (odds ratio [OR] 4.46; 95% CI, 2.35–8.46, *P* = <0.001) and cirrhosis (OR 53.91; 95% CI, 26.49–109.6, *P* = <0.001). Skin rash (51 *vs* 44%) and oral ulcers (45 *vs* 40%) were high in patients receiving SOF-DCV then SOF-VEL.

**Conclusions:**

Overall, the generic SOF+DCV ±RBV and SOF/VEL ± RBV achieved equally high SVR12 rates. However, SOF/VEL+RBV achieved a high SVR rate in cirrhotic patients then SOF+DCV+RBV. Old age and cirrhosis were significant predictors of reduced odds of SVR regardless of the regimen. Furthermore, the regimens were well tolerated in chronic HCV patients.

## Highlights

- The chance of achieving cure was the same whether a person receive SOF+DCV or SOF/VEL in chronic HCV patients of GT3.
- Adding Ribavirin and extending the duration from 12 to 24 weeks enhanced the SVR rates in cirrhotic patients.
- SOF/VEL was more effective and tolerable with less adverse events in chronic and cirrhotic patients then SOF+DCV.

## Introduction

Chronic hepatitis C (CHC) infection is one of the major causes of liver abnormalities and hepatocellular carcinoma (HCC) globally (Baumert et al., 2019). It is estimated that nearly 71 million people suffering from Hepatitis C and among these 3.5–5 million die per year globally (Lim et al., 2018). In the list of Hepatitis C virus (HCV) highest-burden countries, Pakistan ranked 2^nd^ after Egypt with prevalence (4.5–8.2%) (Sievert et al., 2011; Umer and Iqbal, 2016; Organization, 2017). HCV has seven major genotypes, genotype 3a (GT3a) is the most prevalent (69.1%) form in Pakistan followed by GT1 (7.1%), 2 (4.2%), and 4 (2.2%) (Umer and Iqbal, 2016; Khan et al., 2019). The transmission of HCV is mainly driven *via* therapeutic injections, blood transfusion, syringe reuse, surgery, hospitalization, piercing, and shaving from barbers (Umer and Iqbal, 2016; Trickey et al., 2017; Al Kanaani et al., 2018). At present, Pakistan does not have a national hepatitis surveillance system which indicates the importance of HCV as a public health threat in Pakistan (Al Kanaani et al., 2018).

In the past, Interferon (IFN)-based treatment was the only effective treatment option for HCV but it is having a low sustained virological response (SVR) rate (50%) and with many reported unwanted effects (Manns et al., 2001). Hepatitis Control Programs in the country was initiated in 2011 including the “Chief Minister’s Hepatitis Control Programs,” it was reported that successful treatment outcomes were achieved only in 67–74% HCV patients with IFN-based treatment (Qureshi et al., 2013; Ali et al., 2016). Since 2013, the Pakistan Health Research Council is coordinating the hepatitis response at the federal and provincial levels through a “Technical Advisory Group” (TAG). The TAG played a key role in making direct-acting antivirals (DAAs) available in Pakistan at a very low price (Organization, 2017).

The addition of DAAs was a breakthrough in HCV treatment worldwide, these drugs having a function of inhibition in the replication cycle of the hepatitis C virus (Spengler, 2018). The three-drug classes of direct-acting antivirals i.e. inhibitors of NS3/NS4A protease, NS5A complex, and NS5B polymerase was approved by Food and drug administration (FDA). More than 90% of SVR rates can be achieved by drug combinations from these approved three-classes of DAAs (Spengler, 2018).

Regarding the treatment guidelines of HCV, SOF-based DAAs have been included in the “National Chronic Hepatitis C Infection Treatment Guidelines.” Recently Daclatasvir (DCV), which is an HCV NS5A replication complex inhibitor is included in the National Hepatitis Control Program and can be used in combination with SOF for 12 weeks against GT3. Better compliance and successful treatment outcomes achieved with the addition of Daclatasvir at the government-level (Cavalcante and Lyra, 2015; Capileno et al., 2017). Velpatasvir (VEL) is another a pan-genotypic HCV NS5A inhibitor and single-tablet regimens (STRs, Epclusa^®^) for the cure of HCV infection (Link et al., 2019). In Pakistan, the sofosbuvir-velpatasvir (SOF/VEL) combination has been approved for use since March 2018 but not included in the National Hepatitis Control Programs. DAAs are not distributed by any national program. Therefore pharmaceutical companies have strong generic competition (SOF: 14 generic versions, DCV: 4 generic versions, SOF/VEL: 1 generic company filed for US$ 180 in 2017) (Organization, 2018).

DAAs are designed against GT1 of HCV and the clinical trials of DAAs included a limited number of GT3, this raises concerns about the effectiveness of these drugs against GT3. Limited data is available on drug therapy for CHC with SOF+DCV *vs* SOF+VEL and the salvage therapy for GT3. This study aimed to evaluate the antiviral efficacy of generic direct-acting antivirals in government and private tertiary care hospitals.

## Methods

### Study Cohort

It is an observational prospective study, conducted in the gastroenterology departments of multiple hospitals of Pakistan. A total of 1,500 viremic HCV patients were consecutively recruited from January 2019 to January 2020. The inclusion criterion was patients ≥18 years old with chronic HCV infection, patients co-infected with HBV/HCV, patients with cirrhosis, and relapsers of interferon-based or DAA-based therapy without NS5A inhibitor. The exclusion criteria included age <18 years, patients on non-DAA, and patients with incomplete profiles. Fibrosis stages were determined by fibro scan before the enrolment of patients. Patients were divided into two treatment groups: an “easy-to-treat group” included (treatment-naive patients: SOF-DCV for 12 weeks) and a “difficult-to-treat group” included (treatment-experienced patients: SOF-VEL for 12 weeks). The cirrhotic patients were treated for 24 weeks by the addition of RBV in respective groups. All patients in government hospitals were entitled to free baseline testing (hematological tests, biochemical tests, genotyping, quantitative PCR, and the Fibro scans) at the expense of the government. However, Private hospital patients performed these tests elsewhere in private labs during and after treatment.

### Recommended Therapeutic Regimens

There were two sofosbuvir-based therapeutic regimens administered to the HCV-GT3 cohort. Generic SOF and DCV were supplied by the government to all government tertiary care hospitals. The relative doses of SOF (400 mg/day) and DCV (60 mg/day) were recommended daily with food for 12 weeks. Ribavirin was administered according to the weight of the patient (1,200 mg/day for > 75kg and 600mg/day for < 75kg). The treating physicians were allowed to modify or discontinue the RBV dose according to the change in hemoglobin. The single-tablet regimen, with a fixed-dose combination of sofosbuvir (400 mg)/velpatasvir (100 mg) were recommended daily for 12 weeks in private tertiary care hospitals.

### Efficacy Endpoints

The treatment efficacy was checked at the end of the treatment (ETR: undetectable HCV-RNA at the completion of treatment) and after 12 weeks of treatment (SVR12: undetectable HCV-RNA at 12 weeks after the completion of treatment). However, cirrhotic patients’ treatment was extended to 24 weeks. Virological failure categories were relapsed patients (HCV-RNA ≥ lower limit of 25 IU/ml during or after treatment) and non-responders (HCV-RNA ≥ lower limit of 25 IU/ml at end of treatment). The quantitative RT-PCR (Qiagen Kit) was used for measuring HCV-RNA.

### Safety Assessments

Safety endpoints included adverse events (AEs), and all patients were included in the safety assessment analysis. All safety assessments were performed according to the protocol of the individual hospital and the recommended guidelines of the HCV program. Laboratory tests for assessments of biochemical and hematological parameters and safety assessments were performed at baseline, EOT, and post-treatment week 12 and 24.

### Pretreatment Assessment Variables

Information about the following variables was acquired from the study cohort.

#### Sociodemographic and Clinical Data of Patients

Age, gender, body mass index (BMI), previous treatment status (naıve or pretreated) and if pretreated, history of previously administered medications. The comorbidities like diabetes, obesity, and hypertension data were assessed with risk factors like smoking, surgery, and blood transfusion.

#### Laboratory Tests and Non-Invasive Tests to Assess Liver Disease Severity

Pretreatment laboratory assessment included complete blood count (CBC) with platelet count and International normalized ratio (INR), Hepatic function panel included aspartate aminotransferase (AST), alanine aminotransferase (ALT), total bilirubin, albumin, and creatinine levels. Before starting antiviral therapy, quantitative HCV RNA (PCR ≥ 80,000 IU/ml: high HCV viral load, PCR < 80,000 IU/ml: low HCV viral load), Hepatitis B surface antigen and genotyping were performed. Transient elastography indicating cirrhosis (Fibro scan stiffness >12.5 kPa), abdominal ultrasound, or prior liver biopsy data was used for the confirmation of compensated cirrhosis (Child-Pugh A).

### Statistics of the Study Cohort

The efficacy and safety analyses were performed on patients received one dose of either treatment regimens. Statistical Package for Social Sciences (SPSS) version 21 software was used for data entry and analysis. Patients’ demographic and laboratory test values were expressed as number (percent) for binary variables and as Mean (± SD) for continuous variables. Baseline data of administered treatment regimen (SOF-DCV *vs* SOF-VEL) were compared. Selected 1,388 patients were those who started treatment during the set time in the selected hospitals. The aim behind comparing these groups was to assess the efficacy of SOF-based combination in HCV-GT3 and the efficacy of the generic SOF-DCV and SOF-VEL in private and government settings. For comparison of two groups, the listed variables were analyzed using Student’s t-test and for comparison of binary variables, a χ^2^ test was used. Univariate and multivariate logistic regression analyses with Wald statistical criteria were performed to identify baseline factors associated with non-SVR. The results were presented as *P* value, adjusted odds ratio (OR) with a 95% confidence interval (CI). A *P* value <0.05 was considered statistically significant for the analysis.

## Results

### Baseline Characteristics of Study Patients

**Figure 1:**
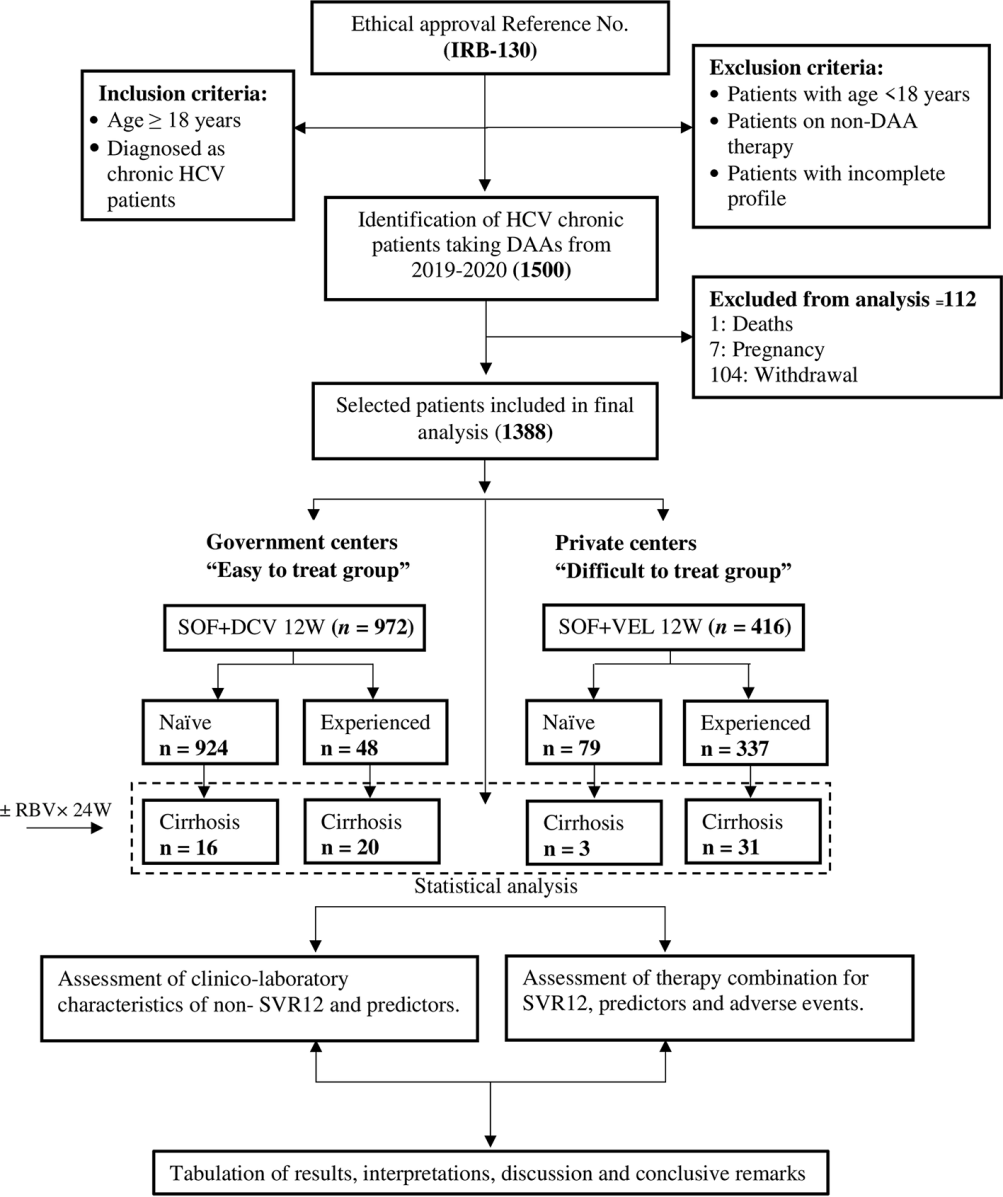
Flow chart of patient’s disposition.

Data were included for 1,388 enrolled patients who received treatment with SOF-DCV (n = 972) by the National Hepatitis Control Program and SOF-VEL (n = 416) by private tertiary care hospitals from January 2019 to January 2020. **Figure 1** shows the patient’s distribution and the numbers included in each regimen.

The mean age was 46.5 ± 13.3 years; most patients were females (52.7%) and treatment-naive (73.3%). Only 5% of patients had compensated cirrhosis so ribavirin was added in their therapy. The frequent comorbidities at baseline included, obesity, diabetes, and hypertension. The risk factors associated with the SVR rate were also analyzed i.e. blood transfusion, surgery, and tobacco smoking. The assignment of therapeutic regimens was based on government and private hospitals including previous treatment history and presence of cirrhosis. “Easy-to-treat group” patients were treated with SOF-DCV for 12 weeks in government hospitals and “difficult-to-treat” patients were treated with SOF-VEL for 12 weeks in private hospitals.

All of the Patients were infected mainly with HCV GT3 irrespective of subtype. As a result of assignment criteria (**Table 1**), more patients treated with SOF-VEL were at old age (53 *vs* 44, *P* < 0.001) and BMI (35 *vs* 25 kg/m^2^, *P* < 0.001). Similarly, the liver enzymes associated with viral infection (ALT, AST) were relatively high in difficult to treat a group of SOF-VEL (AST: 81 *vs* 68 U/L, ALT: 78 *vs* 69 U/L, *P* < 0.001). Significant differences were observed in hemoglobin and platelets levels (*P* < 0.001), diabetes, and obesity groups respectively (8 *vs* 7%, 8 *vs* 9%, *P* < 0.001) and in surgery (8 *vs* 7%, *P* 0.03).

**Table 1:** Baseline demographics and clinical characteristics of study patients (n = 1,388).

Parameter	SOF-VEL(n = 416)	SOF-DCV(n = 972)	*P* value
Age (y), M ± SD	52.9 ± 12.5	43.7 ± 12.6	**<0.001**
Male, n (%)	207 (49.8)	449 (46.2)	
BMI (kg/m^2^)	34.6 ± 6.9	25.2 ± 1.7	**<0.001**
Treatment experienced, n (%)	337 (81)	48 (5)	
Cirrhosis, n (%)	34 (8)	36 (4)	
**Laboratory data**			
HB (g/dL) M ± SD	13.2 ± 2.2	12.8 ± 2.2	**<0.001**
WBCs (×10^9^/L) M ± SD	7.5 ± 1.9	7.6 ± 2.4	
PLT (×10^9^/L) M ± SD	245.8 ± 98	217.4 ± 100	**<0.001**
AST (ULN: 40 U/L) M ± SD	81.4 ± 40.7	68 ± 32.9	**<0.001**
ALT (ULN: 40 U/L) M ± SD	78.23 ± 65.5	69.09 ± 63.2	**<0.001**
Albumin (g/L) M ± SD	41 ± 16.9	39.9 ± 3.4	
Creatinine (mg/dl) M ± SD	98.53 ± 37.2	98.61 ± 41	
TBR (μmol/L) M ± SD	11.59 ± 9.8	11.25 ± 3.4	
INR M ± SD	1.3 ± 0.2	1.2 ± 0.1	
HCV PCR log10	6.5 ± 0.9	6.1 ± 0.8	
**Comorbidities**			**<0.001**
Diabetes, n (%)	31 (7.5)	72 (7.4)	
Obesity	32 (7.7)	76 (7.8)	**<0.001**
Hypertension, n (%)	85 (20)	106 (11)	
HBsAg, n (%)	12 (3)	6 (0.6)	
**Risk factors** Blood transfusion, n (%)	118 (28)	247 (25)	
Surgery, n (%)	32 (7.7)	72 (7.4)	0.03
Tobacco smoking, n (%)	143 (34.4)	263 (27)	

Data are presented M (± SD) or n (%). SOF, sofosbuvir; VEL, velpatasvir; DCV, daclatasvir; BMI, body mass index; HB, hemoglobin; WBC, white blood cell; PLT, platelets; AST, aspartate transaminase; ALT, alanine transaminase; TBR, total bilirubin; INR, international normalized ratio; HBsAg, hepatitis B surface antigen.

### Treatment Efficacy

Overall, SVR at 12 weeks was achieved by 95.5% of patients in this cohort, while 96.8% of patients achieved EOT response. After the end of treatment, 1.5% of patients relapsed. SVR12 rates were high in SOF-VEL regimen receiving patients than SOF-DCV regimen receiving patients [94.7% (95% CI, 94.3–94.9) *vs* 94.4% (95% CI, 94.2–94.8) *P* = 0.04] (**Table 2**). More patients treated with SOF-DCV in government hospitals discontinued therapy than those treated with SOF-VEL in private hospitals (5 *vs* 1.2%, *P* < 0.001). However, the nonresponse rate was more among those patients treated with SOF-DCV than SOF-VEL (4.1 *vs* 3.8%, *P* = 0.05). Relapse rates were similar in both groups of SOF-VEL and SOF-DCV (*P* = 0.01; **Table 2**).

**Table 2:** Assessment of treatment efficacy among studied patients (n = 1,388).

Parameter	SOF/VEL(n = 416)	SOF-DCV(n = 972)	^*^P value
**End of treatment response**			
Number (%)	403 (96.9)	940 (96.7)	**0.02**
95% CI	(96.0–96.9)	(96.5–97.2)	
**SVR12 rates**			
SVR12, n (%)	394 (94.7)	918 (94.4)	**0.04**
95% CI	(94.3–94.9)	(94.2–94.8)	
Non-SVR12, n (%)	22 (5.3)	54 (5.6)	
**Non-SVR12 groups (%)**			
Relapsers (% of patients with end of treatment)	6 (1.48)	14 (1.48)	**0.01**
Nonresponder	16 (3.8)	40 (4.1)	**0.05**

*SOF-VEL vs. SOF-DCV. Bold values represent statistical significance.

As compared to those who did not achieve SVR12 (n = 76), patients who achieved SVR12 (n = 1,312) were younger (P < 0.001), with lower ALT (P < 0.03), lower viral load (P < 0.001), and had higher levels of white blood cells (P < 0.03; **Table 3**).

**Table 3:** Comparison between baseline characteristics of SVR *vs* Non-SVR patients.

Parameter	Non-SVR12 (N = 76)	SVR12 (N = 1,312)	*P* value
Age (y), M ± SD	58.62 ± 16.8	45.80 ± 12.7	**<0.001**
**Age group**			
18–59 n (%)	37 (49)	1,090 (83)	
≥60 n (%)	39 (51)	222 (17)	
**Gender**			
Male, n (%)	30 (39.5)	626 (47.7)	
Female, n (%)	46 (60.5)	686 (52.3)	
BMI (kg/m2), M ± SD	27.63 ± 4.7	28.08 ± 5.9	
**BMI**			
<30, n (%)	54 (71)	950 (72)	
>30, n (%)	22 (29)	362 (28)	
**Treatment status**			
Naive, n (%)	30 (39.5)	973 (74)	
Experienced, n (%)	46 (60.5)	339 (26)	
**Treatment arm**			0.04
SOF/DCV, n (%)	54 (71)	918 (70)	
SOF/VEL, n (%)	22 (29)	394 (30)	
**Cirrhosis**			
Present, n (%)	10 (24)	60 (4.5)	
Absent, n (%)	32 (76)	1,286 (96)	
**Comorbidities** Diabetes, n (%)	23 (30)	80 (6)	
Obesity, n (%)	23 (30.3)	85 (6.5)	
Hypertension, n (%)	58 (76)	144 (11)	
HBsAg, n (%)	1 (1.3)	17 (1.3)	**<0.001**
**Risk factors** Tobacco smoking, n (%)	32 (42)	374 (29)	
Surgery, n (%)	22 (29)	82 (6)	
Blood transfusion, n (%)	46 (60.6)	319 (24)	
**Laboratory data** HB (g/dl) M ± SD	13.03 ± 1.9	12.98 ± 2.2	
WBCs (×10^9^/L) M ± SD	7.04 ± 2.0	7.62 ± 2.3	0.03
PLT (×10^9^/L) M ± SD	208.9 ± 81	226.9 ± 102	
AST (ULN: 40 U/L) M ± SD	69.2 ± 35	72.2 ± 36	
ALT (ULN: 40 U/L) M ± SD	87.9 ± 67	70.9 ± 63	0.03
Albumin (g/L) M ± SD	39.7 ± 6	40.3 ± 9	
Creatinine (mg/dl) M ± SD	99.9 ± 25	98.5 ± 40	
INR M ± SD	1.3 ± 0.1	1.2 ± 0.1	
TBR (μmol/L) M ± SD	10.8 ± 5	11.4 ± 6	
HCV PCR log10	6.2 ± 3	6.1 ± 4	**<0.001**

Bold values represent statistical significance.

Seventy-four percent (56/76) of patients who did not achieve SVR12 was on-treatment non-responders and 26% patients relapsed after the end of treatment. Baseline clinical and laboratory parameters of patients who failed treatment (non-responders and relapsers) are shown in **Table 4**. Significant differences were observed in BMI >30 (P = 0.01), treatment arm (P = 0.01) and HCV PCR log10 (P = 0.02).

**Table 4:** Baseline characteristics of virological failures (n = 76).

Parameter	Relapsed (n = 20)	Nonresponder (n = 56)	*P* value
Age (y), M ± SD	61.2 ± 10.2	57 ± 18.6	
Male, n (%)	7 (35)	23 (41)	
BMI >30, n (%)	6 (30)	16 (29)	**0.01**
Treatment experienced, n (%)	13 (65)	33 (59)	
Cirrhosis, n (%)	17 (85)	27 (48)	
**Treatment arm**			**0.01**
SOF/DCV, n (%)	14 (70)	40 (71.4)	
SOF/VEL, n (%)	6 (30)	16 (28.6)	
**Laboratory data**			
HB (g/dl) M ± SD	13.1 ± 1.6	13.0 ± 2	
WBCs (×10^9^/L) M ± SD	7.6 ± 2.6	6.8 ± 1.8	
PLT (×10^9^/L) M ± SD	203 ± 77	211 ± 83	
AST (ULN: 40 U/L) M ± SD	69.9 ± 34.2	68.9 ± 36.3	
ALT (ULN: 40 U/L) M ± SD	87.32 ± 68.4	88.2 ± 68.5	
Albumin (g/L) M ± SD	39.6 ± 3.5	39.8 ± 6.3	
Creatinine (mg/dl) M ± SD	99.03 ± 25	100 ± 26	
INR M ± SD	1.2 ± 0.1	1.1 ± 0.1	
TBR (μmol/L) M ± SD	9.44 ± 3.4	11.29 ± 5.6	
HCV PCR log10	6.2 ± 0.3	6.4 ± 0.9	**0.02**
**Comorbidities**			
Diabetes, n (%)	13 (65)	10 (18)	
Obesity, n (%)	13 (65)	10 (18)	
Hypertension, n (%)	20 (100)	38 (67)	
HBsAg, n (%)	1 (5)	0 (0.0)	
**Risk factors**			
Blood transfusion, n (%)	19 (95)	27 (48)	
Surgery, n (%)	12 (60)	10 (18)	
Tobacco smoking, n (%)	19 (95)	13 (13)	

Bold values represent statistical significance.

### Baseline Predictors of Sustained Virological Response at 12 Weeks

In multivariate binary logistic regression analysis, the treatment regimen was not a significant predictor of odds of SVR. However, age (OR, 4.46; 95% CI, 2.35–8.46, *P* = <0.001) and cirrhosis (OR, 53.91; 95% CI, 26.49–109.6, *P* = <0.001) had statistically significant association with non-SVR. Patients with age above 60 years exhibited greater non-SVR rates. Similarly, those patients who had cirrhosis were less likely to achieve higher SVR in 12 weeks **(****Table 5****)**.

**Table 5:** Multivariate logistic regression analysis for baseline predictors of non-SVR.

Variable	Non-SVR12 (No. %)	Univariate analysisOR (95% CI)	P-value	Multivariate analysisOR (95% CI)	P-value
Age >60 yr.	37 (49)	6.68 (4.14–10.79)	**<0.001**	4.46 (2.35–8.46)	**<0.001**
Male gender	30 (40)	0.71 (0.44–1.14)	**0.16**	0.94 (0.52–1.70)	0.84
Treatment experienced	46 (61)	4.40 (2.73–7.08)	**<0.001**	1.26 (0.65–2.43)	0.47
HCV PCR >800,000	32 (42)	3.07 (1.91–4.94)	**<0.001**	0.90 (0.44–1.83)	0.78
ALT >ULN	59 (78)	1.56 (0.89–2.71)	**0.11**	1.38 (0.70–2.73)	0.34
AST >ULN	53 (70)	0.82 (0.49–1.36)	0.44		
Albumin <3.5 g/L	4 (5.3)	1.08 (0.38–3.05)	0.88		
Total bilirubin >ULN	3 (4)	1.41 (0.42–4.70)	0.57		
WBC <4×10^9^/L	2 (3)	1.83 (0.42–8.04)	0.41		
Hemoglobin <10 g/dl	2 (2.6)	0.38 (0.09–1.57)	**0.18**	0.74 (0.15–3.53)	0.70
Platelets <150 (×10^9^/L)	15 (20)	0.96 (0.53–1.7)	0.89		
Creatinine >ULN	4 (5)	1.40 (0.49–3.99)	0.52		
Compensated cirrhosis	44 (58)	68.0 (37.3–123.7)	**<0.001**	53.91(26.49–109.6)	**<0.001**

Bold values represent statistical significance. P-value < 0.25 in the univariate analysis were included in the multivariate analysis.

### Safety and Tolerability

**Table 6** describes the relationship between treatment arm and adverse events (AEs) reported in on-treatment patients during the study period. A significant association was found between treatment regimen and adverse events (skin rash, 49% and oral ulcers, 43%). Skin rash (51 *vs* 44%) and oral ulcers (45 *vs* 40%) were high in patients taking SOF-DCV then SOF-VEL respectively.

**Table 6:** On-treatment adverse events.

Patients, n. (%)	SOF+VEL(n = 416)	SOF+DCV(n = 972)	Total(n = 1,388)	P value
Headache	241 (58)	660 (70)	901 (65)	
Nausea	82 (20)	213 (23)	301 (30)	
Anemia	158 (38)	392 (40)	550 (40)	
Abdominal pain	123 (30)	342 (35)	465 (35)	
Myalgia	123 (48)	341 (55)	734 (53)	
Dizziness	46 (11)	117 (12)	163 (12)	
Diarrhea	86 (21)	240 (25)	326 (24)	
Fatigue	66 (16)	281 (29)	347 (25)	
Skin rash	183 (44)	496 (51)	679 (49)	**<0.001**
Oral ulcers	165 (40)	433 (45)	597 (43)	**<0.001**

Bold values represent statistical significance.

## Discussion

DAA’s development has dramatically revolutionized the treatment of HCV. These therapeutic regimens achieve higher rates of SVR and limit the progression of liver cirrhosis. IFN based therapy against HCV treatment has been ceased around the globe and DAAs-based therapy is progressively exceeding (Spengler, 2018). There is a drastic decline in the prices of DAAs due to its generics availability in 101 developing countries (Hill et al., 2016). Though, scientific assessment and validation are required for verifying the efficacy and safety of these generics. For the treatment of HCV infection at large-scale, it would be judicious to analyze the prevailing experience with these therapeutic regimens in real-life settings among all groups of HCV patients.

We report here a real‐world experience with two groups of generics in government and private settings. In this large real-world population, patients were distributed in an “easy to treat group” treated in government hospitals by SOF+DCV and “difficult to treat group” were treated in private hospitals by SOF/VEL. Easy to treat group included mostly treatment naïve patients and difficult to treat group included mostly treatment-experienced and cirrhotic patients with total bilirubin >1.2 mg/dl, albumin <3.5 g/dl, platelets <150 × 10^3^/L, and viral load >800,000 IU/ml. Overall SVR rates were 95.5% treated with either SOF+DCV or SOF/VEL. The SVR12 rates of SOF+DCV (94.4%) were comparable to SOF/VEL (94.7%). Clinical trials did not compare directly SOF+DCV and SOF/VEL regimens. However, SOF/VEL has achieved higher SVR rates in clinical trials (Falade-Nwulia et al., 2017) but the present data show that SOF+DCV or SOF/VEL achieve similar SVR rates even among difficult to treat groups. The results of our study are in line with the studies conducted elsewhere (Omar et al., 2018; Belperio et al., 2019).

The European Association for the Study of the Liver (EASL) recommends SOF+DCV ± RBV or SOF/VEL ± RBV (RBV addition dependent upon treatment-experienced and cirrhosis status) for GT3 (Pawlotsky et al., 2018). Similarly, the American Association for the Study of Liver Diseases (AASLD) recommends 12 weeks of SOF+DCV or SOF/VEL for treatment-naïve and experienced patients without cirrhosis and addition of RBV for experienced patients with cirrhosis (Chung et al., 2018). We observed overall 97% of SVR rate in treatment-naïve patients (SOF/VEL: 98.7%, SOF+DCV: 96.9%, *P* = 0.04). Similar findings have been reported by the Meta-analysis of Pisaturo et al. where the prevalence of SVR12 by SOF/VEL in treatment naïve patients was 98% (Mariantonietta et al., 2019). He analyzed 4,907 patients from 16 studies. Among 4,907 patients, 1,431 patients were of GT3 with 96% (95% CI: 93–99%) prevalence of SVR12. In comparison to our response rate with SOF+DCV, similar outcomes are reported in other studies (Nelson et al., 2015; Welzel et al., 2016; Goel et al., 2017; Hézode et al., 2017; del Rio-Valencia et al., 2018; Belperio et al., 2019). These findings supported that sustained virological responses are comparable between SOF+DCV and SOF/VEL. It shows the use of RBV did not improve the SVR12 rate. Moreover, Cornberg et al. reported the adverse events associated with the use of RBV (Cornberg et al., 2017).

In the current study, the overall SVR12 rate was 88% in treatment-experienced patients. Numerically, higher SVR rates (93.8%) have been observed in treatment-experienced patients receiving SOF/VEL then SOF+DCV due to a smaller number of pretreated patients with cirrhosis status. This is in agreement with the results published by Belperio et al. (2019) and Butt et al. (2020). Similarly, in our case RBV was added only in cirrhotic cases (n = 70, 26/44), the rest of the patients were treated without RBV due to its adverse effects. SVR12 rates were generally lower in cirrhotic patients, with a history of decompensation. The use of RBV and extension in treatment duration from 12 to 24 weeks increased the SVR rates from 77 to 88 and 83% respectively (SOF/VEL; 26/34 to 30/34, SOF+DCV; 0/36 to 30/36). The results of our study are similar to the study of Belperio et al. where extending treatment duration in both groups of treatment arm increased the SVR rate (Belperio et al., 2019). Similarly, Markus et al. and Michael et al. assess the addition and duration of RBV in cirrhotic patients which is in line with our study results (Curry et al., 2015; Cornberg et al., 2017). The present data indicate that SOF/VEL (88%) and SOF+DCV (83%) achieved higher SVR rates even among cirrhotic patients.

Cure rates >90% have been reported by many studies using different combinations of DAAs in chronic HCV patients (Del Bello et al., 2016; Welzel et al., 2016; Zhu et al., 2016; Belperio et al., 2019; Mushtaq et al., 2020). Given the high SVR rates with DAAs, virological failure cases were relatively low due to effective treatment strategies and the right drug combinations (Buti et al., 2015; Benítez-Gutiérrez et al., 2016; Soriano et al., 2016; Mushtaq et al., 2020).

However, initial real-world results supported these findings, but the efficacy tends to be lower over time mainly due to the predictors associated with a lower SVR rate. Furthermore, DAAs success have been compromised by doctor’s limited expertise using new DAAs combinations (Dieterich et al., 2014; Jensen et al., 2014; Backus et al., 2015; Del Bello et al., 2016; Soriano et al., 2016; Sulkowski et al., 2016; Zhu et al., 2016; Arias et al., 2017). Similarly, in our study, patients who failed treatment were 6% (76/1388). Among them 74% (56/76) of patients who did not achieve SVR12 were non-responders and 26% (20/76) were relapsed after the EOT. The primary nonresponse occurred slightly more among those treated with SOF-DCV than SOF-VEL. However, relapse rates were the same in both groups. The reason could be the cirrhotic patients added in easy to treat group rather in difficult to treat group and increased the non-SVR rate. The SVR rate was later increased to 88% (SOF/VEL) and 83% (SOF+DCV) by the addition of RBV for 24 weeks. Since, the regimen was not found to be a significant predictor of SVR, which is in agreement with the guidelines of EASL’s and AASLD for the recommendations of SOF+DCV and SOF/VEL as a therapeutic regimen against HCV-GT3.

Furthermore, SVR-associated predictors are not uniform throughout the clinical trials and real-world studies, which is challenging to make comparisons between the efficacies of different DAAs combinations. To date, baseline variables (liver cirrhosis, prior-treatment experience, infection with HCV GT1 or GT3, high viral load, elevated liver enzymes, and the natural polymorphisms in non-structural genes of HCV that reduce drug susceptibility) are found to be associated with lower SVR rates (Buti et al., 2015; Lontok et al., 2015; Benítez-Gutiérrez et al., 2016; Belperio et al., 2019; Mushtaq et al., 2020). However, Sulkowski et al. found that SVR12 rates did not vary after the analysis of several factors such as sub-genotyping, IL28 phenotype, RBV use, race, and treatment failure with protease inhibitors (first-generation) (Sulkowski et al., 2014). In our multivariate analysis, the regimen was not a predictor of SVR. Age was associated with a significant 5% reduced odds of SVR and cirrhosis was associated with 54% reduced odds of SVR12. The older and cirrhotic patients were less likely to achieve higher SVR12. These findings are in agreement with the results of the following studies (Werner et al., 2016; Sood et al., 2017; Belperio et al., 2019).

Eradication of HCV around the globe is possible through the use of these effective generic DAAs (Freeman et al., 2016). In our study cohort, these generic DAAs were found to be safe and well-tolerated. The most common AEs were skin rash and oral ulcers which is comparable with our previous study findings where skin rash and oral ulcers were among major side effects (Mushtaq et al., 2020). A study from Egypt reported skin rash and Pakistan reported oral ulcers using generic SOF+DCV (Hill et al., 2016; Umar et al., 2018). However, no study reported so far, the skin rash and oral ulcers by the use of generic SOF/VEL.

## Conclusion

Summing up the collective findings of the study we may infer that generic DAAs (SOF+DCV and SOF/VEL) are equally highly effective for CHC patients of GT3. The overall cure rates (SVR12) were 95.5%, whether a person received SOF+DCV in government hospitals or SOF/VEL in private hospitals. These findings support the existing guidelines for the treatment of GT3 with either SOF+DCV or SOF/VEL. The SVR rates were potentially improved in pretreated and cirrhotic patients treated either with SOF+DCV± RBV or SOF/VEL ± RBV for 24 weeks. The cure rate was lowest in old (>60 years) and cirrhotic patients. It strongly encourages the early diagnosis and treatment of such patients. These therapeutic regimens were safe and equally tolerable with mild adverse effects of skin rashes and oral ulcers.

### Study Limitations

In this study the viral factors i.e. Resistance-Associated Substitutions (RAS) are not assessed for the efficacy of the therapeutic regimens. Furthermore, there was a lack of genotype diversity and other DAAs combinations.

## Data Availability Statement

The raw data supporting the conclusions of this article will be made available by the authors, without undue reservation, to any qualified researcher.

## Ethics Statement

The studies involving human participants were reviewed and approved by the ethical review board of Rawalpindi Medical University & Allied Hospitals (Holy Family Hospital, Benazir Bhutto Hospital and District Headquarter Hospital) and National University of Sciences and Technology (IRB-130). All procedures performed in studies involving human participants were in accordance with the ethical standards of the institutional and/or national research committee and with the 1964 Helsinki declaration and its later amendments or comparable ethical standards. The patients/participants provided their written informed consent to participate in this study.

## Author Contributions

All authors contributed to the article and approved the submitted version. SMu and AmK made substantial contributions to the acquisition and analysis of the data. SMu drafted the manuscript and TA, AmK, and SMa were involved in critical revision for important intellectual content. The study is supervised by SMa.

## Conflict of Interest

The authors declare that the research was conducted in the absence of any commercial or financial relationships that could be construed as a potential conflict of interest.

## Abbreviations

DAAs, direct acting antivirals; SOF, sofosbuvir; DCV, daclatasvir; VEL, velpatasvir; RBV, ribavirin; BMI, body mass index; HCV, hepatitis C virus; DM, diabetes mellitus; HTN, hypertension; EOT, end of treatment; SVR, sustained virological response; IFN, interferon; HB, hemoglobin; WBCs, white blood cells; PLT, platelets; TBR, total bilirubin; INR, international normalized ratio; AST, aspartate aminotransferase; ALT, alanine aminotransferase; RAS, resistance-associated substitutions; CHC, chronic hepatitis C; EASL, European Association for the Study of the Liver; AASLD, American Association for the Study of Liver Diseases.
